# Charcot Neuroarthropathy: Current Surgical Management and Update. A Systematic Review

**DOI:** 10.3389/fsurg.2022.820826

**Published:** 2022-03-08

**Authors:** Mohd Yazid Bajuri, Shir Lee Ong, Srijit Das, Isa Naina Mohamed

**Affiliations:** ^1^Department of Orthopaedic and Traumatology, Faculty of Medicine, Universiti Kebangsaan Malaysia Medical Centre, Kuala Lumpur, Malaysia; ^2^Department of Human Clinical Anatomy, Faculty of Medicine, Universiti Kebangsaan Malaysia Medical Centre, Kuala Lumpur, Malaysia; ^3^Department of Pharmacology, Faculty of Medicine, Universiti Kebangsaan Malaysia Medical Centre, Kuala Lumpur, Malaysia

**Keywords:** Charcot, neuroarthropathy, hindfoot, surgical reconstruction, fixation

## Abstract

**Background:**

Charcot neuroarthropathy of the ankle and the hindfoot is a complex clinical entity with a high risk of amputation. Charcot neuroarthropathy limb reconstruction has been proposed as a limb-salvaging procedure. However, there was a lack of information on the various available reconstruction methods, including the outcomes and complications. The present study aimed to evaluate the current literature and update on the trends regarding the surgical management of Charcot neuroarthropathy of the ankle and the hindfoot.

**Methods:**

All data published from January 2010 to January 2020 that investigated the methods of fixation and their respective outcomes for the surgical reconstruction in Charcot neuroarthropathy were analyzed. The union rate, amputation rates, and complications associated with these techniques were taken for statistical analysis.

**Results:**

A total of 16 studies fit the inclusion criteria of this study, with four Level-III studies and 12 Level-IV studies were included. Ten studies utilized internal fixation only; five used a combination of internal fixation and circular external fixator, whereby there are three comparative studies between internal and external fixations, and two studies applied combined technique of internal and external fixations (hybrid fixation). One study describes the usage of circular external fixation only.

**Conclusions:**

The use of retrograde intramedullary nail as a treatment of choice in the reconstruction of Charcot neuroarthropathy ankle is recommended before an ulcer occurrence. Hydroxyapatite (HA)- coated screws are recommended for the locking mechanism to prevent migration in Charcot neuroarthropathy due to poor bony quality. Hybrid fixation is recommended for reconstruction in a condition of ulceration and more complex deformity as it provides a higher rate of limb salvage with less soft tissue irritation.

## Introduction

Ankle joint complex is a modified synovial hinge joint, which consists of talocalcaneal, tibiotalar, and trans-tarsal joint. Congruency of the bone and ligament within the ankle gives a high level of compatibility and creates a high degree of stability, while less susceptible to degenerative processes if compared with other joints, hips or knees (1). Ankle joint complex bears a force of approximately five times body weight during a stance phase in normal gait, and up to 13 times body weight during activities such as running. Mobility of the ankle plays an important role in posture control as well (2).

Charcot neuroarthropathy at the hindfoot and ankle level is more challenging compared to those involving midfoot as the deformities are often multiplanar (3). Changes of gait in Charcot neuroarthropathy limbs are caused by alteration of the biomechanics and proprioception of the involved ankle (1). Malalignment in the Charcot limb is prone to ulceration due to altered plantar pressure, limited soft tissue coverage, and pressure over bony prominence. It is always associated with limb shortening due to bone collapse that is caused by avascular necrosis or a neuropathic fracture (3).

There are several anatomical classifications for Charcot neuroarthropathy based on a destruction pattern to foots and ankles. There are five different types of anatomical destruction and frequency of complications mentioned by Sanders and Frykberg (4) where type I is in forefoot (15%), type II is in tarsometatarsal joints (40%), type III is in naviculocuneiform, talonavicular and calcaneocuboid joints (30%), type IV is in ankle and/or subtalar joint (10%), and, lastly, type V is in the calcaneus (5%) (4). Brodsky classified the disease according to the fourth most commonly affected area in Charcot neuroarthropathy; the most common site is in midfoot (60%), followed by hindfoot (30–35%), and then ankle (9%), lastly, calcaneal (2%) (5). Ankle and the subtalar joint was the 2^nd^ most common site for Charcot neuroarthropathy after the Lisfranc joint, according to Sander, Frykberg, and Brodsky (4, 5).

Besides anatomical classification, Charcot neuroarthropathy can be staged according to its physiological progression and radiological appearance at the described stage. It is started with a development stage where the affected limb was markedly warm, erythematous, and swollen, evidenced by periarticular fracture and bony debride radiologically, followed by a reduction of the sign of inflammation and radiographic appearance osseous resorption in the coalescence stage. Lastly, the limb will be reached to the remodel stage, where consolidation of a fracture and a deformed bone occur without a sign of inflammation (6). The main goal of managing Charcot neuroarthropathy is to achieve an osseous stable, painless, plantigrade foot ulcer-free foot (7, 8). Management of Charcot neuroarthropathy was evolving from amputation traditionally to a reconstructive limb-salvaging procedure. However, there is no existing standard consensus regarding surgical treatment of ankle Charcot neuroarthropathy due to the heterogeneity of the disease entity and clinical presentation. Surgical approaches depend on several factors, such as the location of the deformity, ulceration, infection, stability of an affected joint, and surgeon experience (8). Therefore, a systematic review was done among pieces of literature published from January 2010 to January 2020 regarding the surgical procedures implemented in the ankle Charcot neuroarthropathy reconstruction to gain a more current, comprehensive, and effective reconstruction mode. The objectives of the present study were to analyse the existing literature and update on the current trends of the surgical management of Charcot neuroarthropathy of the ankle and the hindfoot.

## Methodology

**Figure 1:**
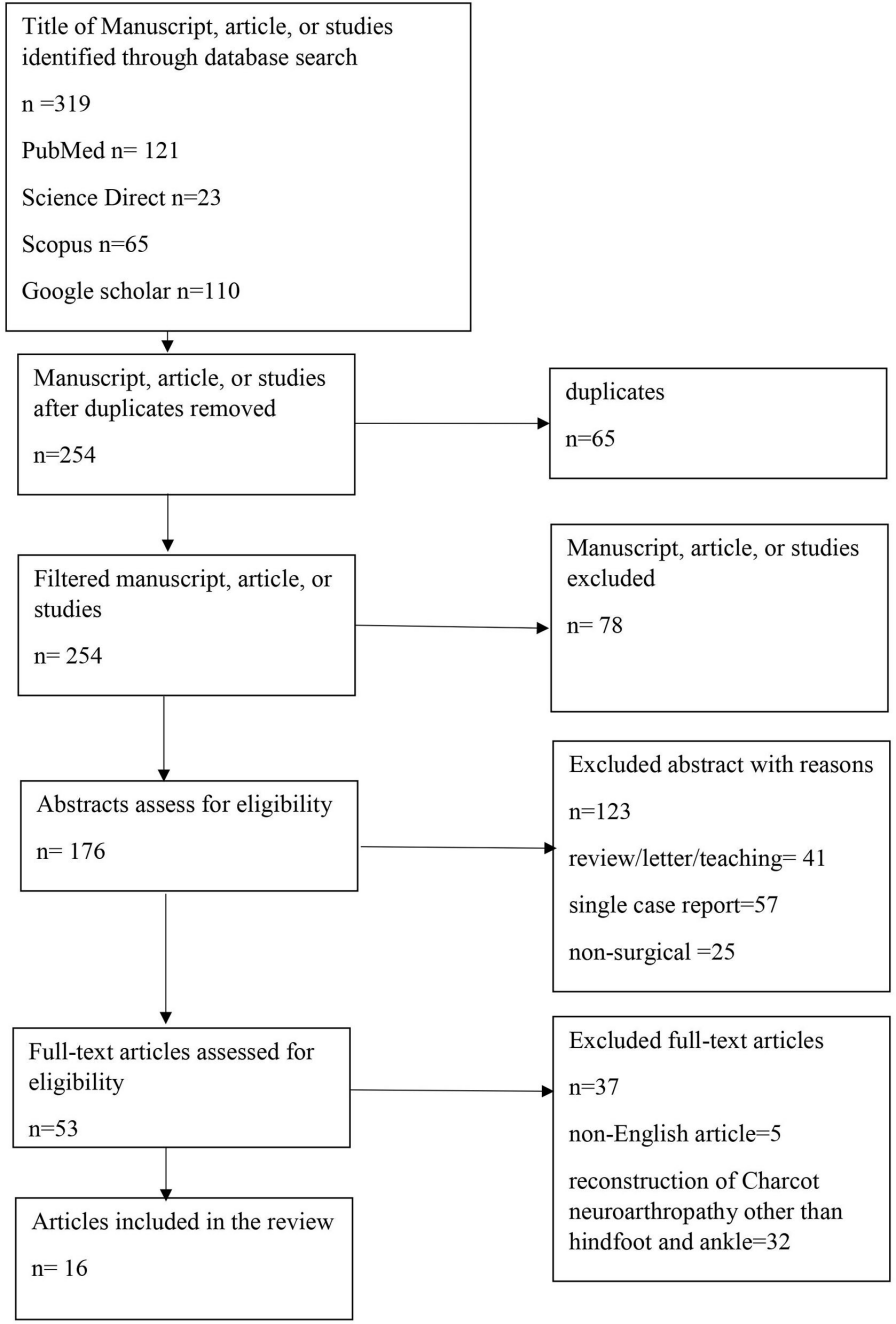
A preferred reporting items for systematic reviews and meta-analyses diagram.

The preferred reporting items for systematic reviews and meta-analysis (PRISMA) checklist was used for the current systematic review (Figure 1). Data were searched through Medline (Ovid, PubMed), Science Direct, Scopus, and Google scholar by using terms: Charcot neuroarthropathy, neuro-osteoarthropathy, osteoarthropathy, neurogenic arthropathy, ankle, hindfoot, surgical, diabetic reconstruction, and fixation. All English-published original papers from January 2010 till January 2020 that comprise of all human or case-control studies, randomized cross-over studies, randomized controlled trials, randomized cross-pilot studies, pre-post-design studies, surgical management at each stage of Charcot arthropathy of ankle or hindfoot, an article in which a long-term outcome post-intervention on Charcot neuroarthropathy is mentioned were included in this study. Papers not published in English, case reports, animal studies, letters to the editor, and review articles were excluded. The terms Charcot arthropathy, neuroarthropathy, neuropathic arthropathy, and neuropathic osteoarthropathy were used interchangeably for this study.

The search was started in May 2019. All the articles were screened independently by the primary author and the second author in three phases: title, abstract, and full-text screening. The number of articles on which all our reviewers agreed in terms of inclusion and exclusion was divided by the total number of double-screened papers to determine inter-observer agreement. Discrepancies among both the first and second authors were resolved by consensus. For full-text assessment and data extraction, all eligible articles follow mutual consensus. Information on demographic of included patients, the method of reconstruction, infection, union, amputation, and hardware complications were counted in this review. The method of application of the technique was extracted among data. The first author collected and screened all included data. The second author independently validated the completed data extraction sheets against the articles.

## Results

Three hundred nineteen reports were yielded from the initial research. Two hundred fifty-four papers remained after duplicates were lifted. Seventy-eight were excluded during title screening, and one 223 out of 176 abstracts were excluded as those were review articles, single case reports, and non-surgical treatment on Charcot neuroarthropathy. The remaining 53 full texts were assessed for eligibility, five non-English published journals were excluded, and 32 were further eliminated because they had included patients who had undergone Charcot neuroarthropathy surgery in a location other than hindfoot and ankle and did not have separate results for the Charcot patients receiving surgery. Therefore, 16 studies were included in the outcome analysis regarding the surgical reconstruction of Charcot neuroarthropathy in the hindfoot and the ankle.

Most of the studies were studies with a level of evidence of IV (twelve out of sixteen) (9–20), and remaining studies of four carry the level of evidence of III (21–24). The present study includes three prospective case series (12, 23, 24), 12 retrospective case series (9–11, 13–19, 21, 22); there is only one retrospective cohort (21) with one therapeutic study (20). The most cited procedures were intramedullary nail and circular external fixation or illizarov external fixation. The table below lists the data summarizing this study based on patient demographics (Table 1) and the outcome of these studies (Table 2).

**Table 1:** A summary of data extraction for a systematic review based on patient demographics.

**Investigator**	**Country**	**Evidence grading (level)**	**Study design and time frame for data collection**	**Sample size and CN classification**	**Participants characteristics**		**Post-operative follow-up (months)**
					**Sex**	**Age (years)**	
Cinar et al. (9)	Turkey	IV	Retrospective case series, 2006–2008	4 patients	2 males, 2 females	53–70 (Mean 63)	12–35 (average=24)
Caravaggi et al. (21)	Italy	III	Retrospective cohort Jan 2001–March 2009	45 patients, Brodsky type 3	27 males, 18 females	56 ±11	62.9 ± 34.19
Chraim et al. (10)	Austria	IV	Retrospective case series Jan 2011–June 2013	18 patients (19 feet) Sander pattern IV (18) Eichenholtz stage III (18)	10 males 8 females	38.5-79.8 (Mean 63.43)	37-70 (mean=46.36)
DeVries et al. (11)	USA	IV	Retrospective case series, July 2003–May 2009	52 patients, Brodsky type 3a	30 males, 22 females	35–85 (Mean 60)	0–66.64 (average = 22.09 ± 17.89)
ElAlfy et al. (12)	Egypt	IV	Prospective case series, Feb 2010–Oct 2013	27 patients	16 males, 11 females	32–75 (Mean 54)	26–45 (mean=31)
El-Mowafi et al. (13)	Egypt	IV	Retrospective case series, Jan 2010–Dec2015	24 patients, Brodsky IIIa	7 males, 17 females	43–62 (mean 50.7)	24–98 (36.4 ± 5.8)
Emara et al. (14)	Egypt	IV	Retrospective case series, 2011–2016	42 patients	31 males, 11 females	38–59 (Mean 49.6)	12
Ettinger et al. (15)	Germany	IV	Retrospective case series Jun 2010–March 2015	58 patients	33 males 25females	26–81 (mean 59.1)	12–57 (mean = 31.3)
Harkin et al. (16)	USA	IV	Retrospective case series 2004–2017	56 patients	Not specified	57.9 ± 9.6	13.2–168
Pinzur et al. (17)	USA	IV	Retrospective case series. No specific duration mention	73 patients	41 males, 32 females	31–76 (means 57.9)	12
Richman et al. (22)	USA	III	Retrospective comparative series, 1999–2014	27 patients	21 males, 16 females	56–57.6	43.2 (retrograde nail), 26.4 (ring fixator)
Siebachmeyer et al. (18)	UK	IV	Retrospective case series Jan 2008–April 2013	20 patients (21 feet)	12 males, 8 females	46-83 (means 62.6)	8-54 (mean = 26)
Sundarajan et al. (23)	India	III	Prospective study July 2007 –Dec 2012	33 patients, Eichenholtz, stage I (9), stage II (13), stage III (11)	19 males, 14 females	41–76 (means 58)	44
Vasukutty et al. (24)	UK	III	Prospective case series, Jun 2008–Sept 2015	40 patients (42 feet)	20 males, 20 females	33–82 (means 59)	12
Kuharajan et al. (19)	Malaysia	IV	Retrospective case series, Jan 2011–Jun 2016	16 patients, Eichenholtz, stage I (0), stage II (0), stage III (16)	4 males, 12 females	20–71 (means 58.1)	At least 6
Pawar et al. (20)	USA	IV	Therapeutic study (2008–2010)	Eichenholtz stage I (1), stage II (0), stage III (3)	4 males, 1 female	46–82	12–24 (average=18)

**Table 2:** A summary of data extraction for a systematic review based on a patient outcome.

**Investigator**	**Procedure**	**Surgical technique**	**Additional procedure**	**Surgeries analyzed**	**Infection**	**Hardware complication**	**Amputation**	**Union**
Cinar et al. (9) (Turkey)	Tibiocalcaneal arthrodesis	Posterior blade plate	Subtotal talectomy; Total talectomy	4	0%	0%	0%	75 % (3/4)
Caravaggi et al. (9) (Italy)	Tibiocalcaneal arthrodesis	Retrograde intramedullary nail	Nil	45	22.2% (10/45)	22.2% (10/45)	8.88% (4/45)	82.2% (37/45)
Chraim et al. (10) (Austria)	Tibiotalocal caneal arthrodesis	Retrograde intramedullary nail	Talectomy	19	15.78% (3/19)	21% (4/19)	15.78% (3/19)	79% (15/19)
DeVries et al. (11) (USA)	Ankle arthrodesis and hindfoot	Retrograde intramedullary nail (45)	Posterior lengthening; Talectomy; Tarsal fusion; Tendon transfer	45	51.1% (23/45)	74.2% (23/31)	22.2% (10/45)	71.1% (32/45)
		Retrograde intramedullary nail and Circular external fixation (7)		7	43.8% (3/7)	43.8% (3/7)	28.6% (2/7)	71.4% (5/7)
ElAlfy et al. (12) (Egypt)	Tibiotalar arthrodesis	Ilizarov external fixation (14)	nil	27	21.4% (3/14)	21.4% (3/14)	0%	85.7% (12/14)
		Retrograde intramedullary nail (13)			7.6% (1/13)	15.8% (2/13)	0%	76.9% (10/13)
El-Mowafi et al. (13) (Egypt)	Tibiotalar arthrodesis	Combine ilizarov external fixation and retrograde Intramedullary nail	Nil	24	33.3% (8/24)	8.3 (2/24)	0%	91.7% (22/24)
K.M. Emara et al. (14) (Egypt)	Tibiotalocalcaneal arthrodesis	retrograde Intramedullary nail	Bone graft	42	14.3% (6/42)	19% (8/42)	0%	33.3% (14/42)
S.Ettinger et al. (15) (Germany)	Tibiocalcaneal arthrodesis; Tibiotalocalcaneal arthrodesis	retrograde Intramedullary nail	Talectomy	38	5.2% (3/58)	0%	5.2% (3/58)	100% (38/38)
		ilizarov external fixation		20				84.2% (16/20)
Harkin et al. (16) (USA)	Tibiotalar arthrodesis; Tibiocalcaneal arthrodesis	ilizarov external fixation or retrograde Intramedullary nail	nil	56	46.4% (26/56)	8.9% (5/56)	14.3% (8/56)	87.5% (49/56)
Pinzur et al. (17) (USA)		Circular external fixation	Radical resection of infected bone	73	20.5% (15/73)	No available	(3/73)	Not available
Richman et al. (22) (USA)	Tibiocalcaneal arthrodesis	Retrograde Intramedullary Nail	Nil	16	37.5% (6/16)	25% (4/16)	6.3% (1/16)	62.5% (10/16)
		external fixation		11	18.2% (2/11)	0%	18.2% (2/11)	63.6% (7/11)
M Siebachmeyer et al. (18) (UK)	Tibiotalar arthrodesis	Retrograde Intramedullary Nail	Midfoot fusion bolt; Locking plate of midfoot; Percutaneous Tendo- Achilles lengthening	22	13.6% (3/22)	27.3% (6/22)	0%	90.1% (20/22)
Sundarajan et al. (23) (India)	Tibiotalocalcaneal arthrodesis	Retrograde Intramedullary Nail	Talonavicular arthrodesis Autogenous iliac bone graft	33	15.2% (5/33)	6.1% (2/33)	9.1% (3/33)	84.8% (28/33)
Vasukutty et al. (24) (UK)	Tibiotalocalcaneal arthrodesis	Hindfoot nail	Percutaneous Tendo- Achilles lengthening; Midfoot fusion; 1st metatarso-phalangeal fusion	42	16.7% (7/42)	14.3% (6/42)	2/3% (1/42)	97.6% (41/42)
Kuharajan et al. (19) (Malaysia)	Tibiotalocalcaneal, arthrodesis	Retrograde hindfoot nail	Nil	16	43.8% (7/16)	6% (1/16)	0%	81% (13/16)
Pawar et al. (20) (USA)	Tibiotalocalcaneal, arthrodesis	Retrograde hindfoot nail	Nil	5	0%	0%	0%	100% (0/5)

There were five studies conducted in the United States (11, 17, 18, 20, 22), three in Egypt (12–14), two in the United Kingdom (18, 24), and one each from Turkey (9), Italy (21), Austria (10), India (23), and Malaysia (19). All data were collected in the range between 3 to 16 years. Overall, 526 patients were included in these studies with ages ranging from 20–85 years old. The number of patients included per study ranges from four to 73. In Maywood, United States, a center recorded the highest single surgeon series with 73 patients (17). Post-operative follow-up among these studies ranges from 6 to 168 months. Most of the neuropathy associated with Charcot among these studies is diabetes mellitus. Other causes of Charcot were also identified through our search: Hansen disease, myelomalacia, idiopathic peripheral neuropathy, spinal stenosis, and spina bifida (19, 23, 24).

Overall, 545 procedures were reported to have surgical reconstruction of Charcot neuroarthropathy of the ankle during the study date. The surgical procedures performed included arthrodesis, talectomy, midfoot reconstruction, radical resection of an infected bone, flap, graft, and tendon Achilles lengthening, and medial column fusion. Internal fixation was discussed in 15 studies with 13 utilize retrograde intramedullary nail (10–12, 14–16, 18–24), one study applied antibiotic coated nail (20), and the others reconstruct *via* a posterior blade plate (9).

The circular external fixation was chosen as a tool for reconstruction in six studies (12, 13, 15–17, 22). The combination of the retrograde intramedullary nail with a circular external fixator (hybrid fixation) was applied for reconstruction in two studies. There were three comparative studies between internal and circular external fixation (12, 15, 22) in which two studies reconstruct *via* the combined technique of internal and circular external fixations (hybrid) (11, 13) with one of the studies comparing isolated nail and a combined approach (11). One study describes the usage of circular external fixators only as a mode of reconstruction (17).

## Discussion

Charcot neuroarthropathy (CN) is a progressing disease that weakens the musculoskeletal system. A pathological fracture over the affected joint may occur under substantial stress, which eventually leads to collapse, re-fracture, and joint destruction (24). Surgical reconstruction of Charcot hindfoot and ankle is preferred in the modern era as the affected limb is often not brace-able due to limb malalignment and bony prominences secondary to the deformity (25). Charcot neuroarthropathy reconstruction aims to produce a stable, shoe-able, painless, and plantigrade foot (7, 8). Patient walking ability was noted to improve significantly post reconstruction (26). Reconstruction frequently fails due to loss of protective proprioception, poor bone quality, and impaired wound healing due to multiple comorbidities associated with Charcot neuroarthropathy. Therefore, a concept of “superconstruct” is warranted in these cases, where fusion is extended beyond the zone of injury; extremity shortening by bone resection is required for adequate reduction without undue tension on soft tissue enveloped *via* using the strongest device applied in maximal mechanical position (27). Arthrodesis is the most common procedure found among studies for Charcot reconstruction. It is beneficial to patients with instability, pain or recurrent ulceration that fails conservative management (28–33). In this review, eight out of 16 studies performed tibiotalocalcaneal arthrodesis to reconstruct Charcot neuroarthropathy of the ankle with 346 cases (63%) recorded (10, 11, 14, 15, 19, 20, 23, 24). Tibiotalocalcaneal arthrodesis is a more common fusion site upon the reconstruction of Charcot neuroarthropathy than Tibiocalcaneal fusion with 197 cases recorded. Other than arthrodesis, we also found bone grafting, talectomy, and posterior tendo-Achilles lengthening commonly performed. A meticulous joint surface preparation aids in successful arthrodesis. Therefore, bone resection (talectomy) was done to realign the ankle and the hindfoot to create a plantigrade foot without tension upon reduction. Bone graft was needed to maintain reconstruction and aids in ossification and consolidation of corrected region post-reconstruction. Soft tissue release (tendo-Achilles lengthening) reduces tension upon reconstruction and peak plantar pressure, preventing ulceration post-reconstruction and improving overall walking ability (34). Among internal fixations of ankle and hindfoot Charcot neuropathy reconstruction, retrograde intramedullary nail as used in 14 studies with one by Pawar coated the nail with antibiotic; a posterior blade plate was used by Murat for reconstruction. The intramedullary nail is a load-sharing device that allows early ambulation. It provides higher stability for axial compression and torsion than external fixation-illizarov or a circular frame according to a biomechanical study on the comminuted tibia shaft fracture model by Hasenboehler (35); it provides higher stability for axial compression and torsion. Due to the unsatisfactory bone quality of Charcot neuropathy, rigid fixation is mandatory to achieve union post-reconstruction. Intramedullary nail aids in evenly distributing compression force across the fusion side (36). L. Massari also proved that retrograde intramedullary nailing was effective and stiffer internally for tibiotalocalcaneal arthrodesis (37). Of 303 cases of retrograde nails among our data, the union rate was recorded as 83.1% (280/337), whereas 75% of the plates were used—a study by Murat et el. where a posterior angle blade plate was used in cases with previous intervention. The rationale of utilizing a blade plate *via* a posterior approach was to avoid wound complication with an adequate soft-tissue envelop for bone implant-graft coverage (9).

A circular external fixator offers a better bone and soft tissue tolerance without compromising blood supply as thin wires were used upon reconstruction. The construct of a circular external fixator allows axial micromotion of bone fragments during weight-bearing. Bony stabilization can be done by manipulating frame components without soft tissue tensioning (29, 38, 39). There were a total of 157 circular external fixations recorded as the reconstruction of Charcot neuroarthropathy ankle among our searches. Thirty-five cases of the union were recorded among studies that applied external fixation, but two studies do not mention a union rate as the outcome (16, 17). Harkin did not specify the union rate according to the type of the fixation method of their cases, whereas Pinzur does not record bony union post-reconstruction of their patients. Therefore, the union rate of circular external fixation among available studies in Charcot reconstruction was 78% (35/45).

Hybrid construct that is utilized by El-Mowafi to combine intramedullary nail and circular external fixation shows a 100% limb salvage and 91.6% union (13). In the hybrid construct by El-Mowafi, the retrograde nail was unlocked distally. In the hybrid construct by El-Mowafi, the retrograde nail was unlocked distally.JG DeVries applied a similar construct in population with more comorbidities and complex deformity; however, there is 71.4% limb salvage, and a union rate was recorded among these cases (11). El-Mowafi proposed a continuous guided compression provided by circular external fixation across the reconstruction site when the intramedullary nail was unlocked distally, which is a more superior option compared to a fixed intramedullary nail. Both authors believe that circular external fixation provides additional stability upon reconstruction and protection during unintentional weight-bearing post-operation. Additional surgery-frame removal was warranted, which may be the disadvantage of this reconstruction form. Most of the studies that discuss Charcot ankle reconstruction focus on one hardware hindfoot arthrodesis. Hence, in our review, we investigated three studies that compare two or more hardware outcomes. El Alfy et al. compared external fixation with intramedullary nailing (12), as opposed to Richman et al.; the incidence of the complications was higher among the external fixator group than in the intramedullary nailing group (22). The risk of infection and the need for additional surgery were increased upon reconstruction as noted among cases that utilize external fixator to augment an intramedullary nail, compared to intramedullary nail alone by JG DeVries. Common hardware complications revealed among retrograde nails of our records were screw loosening (7.3%) and migration (3.6%). Screw loosening may cause implant or screw fracture, inadequate compression force distribution, and possible osseointegration failure. According to mechanical engineering, cyclic transverse loads were the culprit for the rotational loosening of screws (40, 41). According to Turner, the holding power of the screws was reduced in bones with lower mineral content, such as osteoporotic, by comparing healthy bovine bones and osteoporotic bones (42). As the bone quality of Charcot neuropathy is poor, the screws for reconstruction holding power were low. In a clinical study on hydroxyapatite (HA)-coated screw, the feature of surface roughness and the relative “oversising” of screw design prove to increase the torque of insertion and the purchase of a screw into the bone, which reduces the rate of screw loosening (42, 43). Siebachhmeyer and Vasukutty utilized HA-coated screws after observing screw loosening in a few of their initial cases that used the standard distal locking screw; there were no remaining cases that used HA-coated locking screws that were loosened and migrated (18, 24). However, there were no implant failures noted among cases that applied for a posterior blade plate despite a 50% rate of postoperative infection recorded (9). There were 8 cases of hardware complication recorded among studies reconstructed *via* circular external fixation (11–13, 16). However, no hardware complications were noted in 2 studies from Ettinger and Richman, who applied circular external fixators as tools for Charcot reconstruction (15, 22). Hardware complications were not recorded in the study by Pinzur (17). The amputation rate among collected data reconstructed *via* internal fixation was recorded as 7.17% (22/30) and 9.7% (16/164) among cases with circular external fixation. There was 100% limb salvage in Charcot reconstruction with hybrid fixation (combination of the intramedullary nail and circular external fixation) by El-Mowafi despite bony union that was achieved 91.7% in the series (13). About 71.4% of limb salvage was recorded in DeVries's study, where a retrograde nail was augmented with a circular external fixator (11). Ideal timing for Charcot ankle reconstruction is still a debatable topic among surgeons with various approaches and outcomes recorded. Traditionally, surgical reconstruction of Charcot neuroarthropathy was delayed till a later stage of the disease, but there is a risk of skin breakdown and introduction of the infection to bones and joints due to deformity progression. It was due to the belief that a higher rate of infection, wound healing issue, and implant failure when reconstruction was done during the acute phase of Charcot neuroarthropathy (7, 44–46). Most of the authors reconstruct the Charcot ankle at Stage II or III, according to Eichholtz. Early intervention in the early stage of the disease was suggested by Caravaggi (21) when pressure ulcer over bony prominences had not yet developed. B Elalfy also suggested intervention should be done when the deformity was endangered to the skin with a potential of ulcer occurrence (12). Most of the authors reconstructed the Charcot ankle during the chronic stage of the disease. However, there is no complication recorded post-reconstruction in Stage I Charcot in the Sundararajan study. Time for union is also shorter in those groups of patients than those reconstructing Stages II and III. According to the authors, there is no significant difference across the stages in terms of outcomes and complications (23). This study revealed two different surgical approaches upon reconstruction in the presence of osteomyelitis in Charcot neuroarthropathic ankle by utilizing an antibiotic-coated nail by Pawar (21) and a single stage of resection of infection, deformity correction *via* circular external fixation by Pinzur (17). Common treatment strategy in infected Charcot has been infection control *via* debridement, followed by procedures to achieve osseous union either by external or internal fixation. Pinzur recorded 95.7% limb salvage in the studies, but the circular external fixator was required to be removed after at least 12 weeks, where implant removal was not needed in Pawar study where amputation was not recorded. The antibiotic-coated nail provides local antibiotics and can serve as a stable definitive fixator in the infected Charcot neuroarthropathic limb. The limitation of the current study is failure to identify any Levels I and II studies to take note on surgical management on Charcot neuroarthropathy; it is probably due to uniqueness of the disease in each case, making surgical approach divert and a relatively small population of patients treated in this condition to allow a higher level of study to be conducted. Although it is difficult to conduct a prospective randomized-controlled study, such studies would contribute crucial appraisal on various fixation methods in the surgical reconstruction of the Charcot neuroarthropathy ankle. Multicentre studies and registries development can be helpful to achieve adequate patient numbers and increase the strength of the study.

## Conclusion

In conclusion, evidence would suggest a retrograde intramedullary nail as the choice of reconstruction of the Charcot neuroarthropathy ankle before ulcer occurrence. Due to poor bony quality in the Charcot neuropathy ankle, hydroxyapatite (HA)-coated screws are recommended for the locking mechanism to prevent migration. Hybrid fixation is recommended for reconstruction in the condition of ulceration and more complex deformity as it provides a higher rate of limb salvage with less soft tissue irritation.

## Data Availability Statement

The original contributions presented in the study are included in the article/supplementary material, further inquiries can be directed to the corresponding author.

## Author Contributions

MB, SD, IM, and SO: study design, revision and editing of the manuscript, statistical analysis and interpretation of results, and drafting of study protocol. MB and SO: drafting of the initial manuscript. All authors have read and approved the final manuscript.

## Conflict of Interest

The authors declare that the research was conducted in the absence of any commercial or financial relationships that could be construed as a potential conflict of interest.

## Publisher's Note

All claims expressed in this article are solely those of the authors and do not necessarily represent those of their affiliated organizations, or those of the publisher, the editors and the reviewers. Any product that may be evaluated in this article, or claim that may be made by its manufacturer, is not guaranteed or endorsed by the publisher.

## Abbreviations

CN: Charcot neuroarthropathy
HA: Hydroxyapatite
PRISMA: Preferred reporting items for systematic reviews and meta-analysis.
